# Detection of Cancer Mutations by Urine Liquid Biopsy as a Potential Tool in the Clinical Management of Bladder Cancer Patients

**DOI:** 10.3390/cancers14040969

**Published:** 2022-02-15

**Authors:** Nurul Khalida Ibrahim, Ahmed Eraky, Jan Eggers, Tim Alexander Steiert, Susanne Sebens, Klaus-Peter Jünemann, Alexander Hendricks, Corinna Bang, Martin Stanulla, Andre Franke, Claudius Hamann, Christoph Röcken, Norbert Arnold, Laura Hinze, Michael Forster

**Affiliations:** 1 Department of Pediatric Hematology and Oncology, Hannover Medical School, 30625 Hanover, Germany; ibrahim.nurul@mh-hannover.de (N.K.I.); stanulla.martin@mh-hannover.de (M.S.); hinze.laura@mh-hannover.de (L.H.); 2 Institute of Clinical Molecular Biology, Kiel University, 24105 Kiel, Germany; t.steiert@ikmb.uni-kiel.de (T.A.S.); c.bang@ikmb.uni-kiel.de (C.B.); a.franke@mucosa.de (A.F.); norbert.arnold@uksh.de (N.A.); 3 Department of Urology, University Hospital Schleswig-Holstein Campus Kiel, 24105 Kiel, Germany; ahmed.eraky@uksh.de (A.E.); jan.eggers@uksh.de (J.E.); klaus-peter.juenemann@uksh.de (K.-P.J.); claudius.hamann@uksh.de (C.H.); 4 Institute for Experimental Cancer Research, Kiel University and University Hospital Schleswig-Holstein Campus Kiel, 24105 Kiel, Germany; susanne.sebens@email.uni-kiel.de; 5 Department of General Surgery, University Medicine Rostock, 18057 Rostock, Germany; alexander.hendricks@med.uni-rostock.de; 6 Department of Pathology, University Hospital Schleswig-Holstein Campus Kiel, 24105 Kiel, Germany; christoph.roecken@uksh.de

**Keywords:** liquid biopsy, bladder cancer, cancer mutation, urine, molecular diagnostic

## Abstract

**Simple Summary:**

The management of bladder cancer faces multiple challenges concerning the diagnostic and follow-up approaches. The standard diagnostic examination comprises invasive cystoscopy. Urine cytology and recently proposed urine-based biomarkers have been unable to replace cystoscopy, thus prompting calls for improvements. Here, we explore urine liquid biopsy to detect cancer mutations and subsequently evaluate the utility of urine as a suitable specimen for diagnosing bladder cancer. Our results show that the analysis of pre- and postoperative urine with a cost-effective 127-gene panel enables the characterization of tumor mutations. These findings provide cumulative evidence in support of the results of previous studies that testing urine for mutations is a useful strategy to complement the clinical management of bladder cancer patients.

**Abstract:**

The standard diagnostic and follow-up examination for bladder cancer is diagnostic cystoscopy, an invasive test that requires compliance for a long period. Urine cytology and recent biomarkers come short of replacing cystoscopy. Urine liquid biopsy promises to solve this problem and potentially allows early detection, evaluation of treatment efficacy, and surveillance. A previous study reached 52–68% sensitivity using small-panel sequencing but could increase sensitivity to 68–83% by adding aneuploidy and promoter mutation detection. Here, we explore whether a large 127-gene panel alone is sufficient to detect tumor mutations in urine from bladder cancer patients. We recruited twelve bladder cancer patients, obtained preoperative and postoperative urine samples, and successfully analyzed samples from eleven patients. In ten patients, we found at least one mutation in bladder-cancer-associated genes, i.e., a promising sensitivity of 91%. In total, we identified 114 variants, of which 90 were predicted as nonbenign, 30% were associated with cancer, and 13% were actionable according to the CIViC database. Sanger sequencing of the patients’ formalin-fixed, paraffin-embedded (FFPE) tumor tissues confirmed the findings. We concluded that incorporating urine liquid biopsy is a promising strategy in the management of bladder cancer patients.

## 1. Introduction

Bladder cancer is the sixth most commonly diagnosed cancer in men and the tenth in both genders globally [1]. Nearly half of bladder cancer cases are attributed to tobacco smoking [2], followed by occupational exposure to chemicals such as aromatic amines and chlorinated hydrocarbons, which are responsible for 10% of bladder cancer cases [3]. It is not yet clear if family history plays a role in bladder cancer development. Research shows that the risk increases for first- and second-degree relatives, yet genetic predisposition is still under investigation [4,5,6]. Bladder cancer is usually presented with painless macrohematuria [7]. Bladder cystoscopy is the gold standard for diagnosis and follow-up [8]. Follow-up strategies involve repeated cystoscopies associated with the risk of infection and bleeding, and due to its high recurrence, they require patient compliance over a long period of time [7]. Currently, a noninvasive diagnostic tool such as urine cytology is used in clinical practice with variable sensitivity from 16% for low-grade tumors to about 84% for high-grade tumors [9]. Urine cytology has its drawbacks; negative cytology does not exclude malignancy; nonetheless, infections, stone disease, a low number of cells, and subjective interpretation of the test can affect the results drastically [10,11]. Current urine-based biomarkers approved by the US Food and Drug Administration (FDA) for diagnosis and follow-up include UroVysion, NMP22 BladderChek, and NMP22 enzyme-linked immunosorbent assay (ELISA). BTA-TRAK, immunocyte (UCyt+), and BTA-STAT are approved for follow-up only. These markers show high false-positive rates, which limits their use in clinical settings [12]. Urine cytology and urine-based biomarkers do not replace the gold standard, cystoscopy; therefore, the search for reliable tests to use in screening, primary detection, and follow-up of nonmuscle invasive diseases continues. Recent studies in the context of nonmuscle-invasive bladder cancer (NMIBC) have shown that inflammation markers would also be a useful indicator for patient stratification and monitoring, in particular owing to the high recurrence rate of NMIBCs and the role of chronic inflammation [13,14]. For example, studies using peripheral blood measuring the neutrophil-to-lymphocyte ratio have shown evidence of the potential use of the marker in bladder cancer prognostics [15,16].

Next-generation sequencing (NGS) of cell-free DNA (cfDNA) from blood plasma has become the focus of many studies and was recently proposed for the early detection of multiple cancer types. However, its sensitivity in detecting bladder cancer only reaches 35% [17]. Hence, there is growing interest in the utility of urine as a liquid biopsy in bladder cancer. Particularly for a heterogeneous disease such as cancer, liquid biopsy may revolutionize management options for patients through comprehensive molecular characterization. It is also practical for clinical use due to its noninvasive nature and the ease of obtaining and handling the sample compared to traditional tumor biopsies, which can be limited in certain tumor cases. In the context of bladder cancer, urine liquid biopsy has the capability to provide in-depth information on the tumor. In contrast to other tumors and their limited exposure to the blood circulation, urine is in constant contact with the tumor, resulting in the concentrated availability of tumor-related entities in the urine [18]. A recent study reached 52–68% sensitivity using a small NGS panel (10 genes) and could increase sensitivity to 68–83% by adding aneuploidy and telomerase reverse transcriptase gene (*TERT*) promoter mutation detection [19]. This set of three examinations may be too complex for some clinical studies or the clinical routine.

Here, we consider a single examination using just a large NGS panel (127 genes) and find that this simpler set-up gave a similar if not better sensitivity of 91%. Given the options of cfDNA or urothelial cell DNA as input for the panel sequencing, it has been shown that cfDNA amounts in urine are low and variable, making the cfDNA isolation almost as costly as the sequencing [20]. Therefore, we explored whether urothelial cell DNA isolation is more suitable for clinical practice. For this analysis, we included healthy probands to analyze whether (a) gender-based differences and (b) time-of-day variability also apply to urothelial cell DNA.

Our results indicate that urothelial cell DNA may be a promising avenue for analyzing various urothelial tumor entities at specific time points, such as before and after treatments for prognostic or monitoring purposes.

## 2. Materials and Methods

### 2.1. Healthy Volunteers and Patients

Urine from 10 healthy volunteers and 12 bladder cancer patients was studied. The healthy volunteers consisted of 5 males and 5 females. The bladder cancer patients comprised 7 males and 5 females who underwent cystectomy, nephroureterectomy, or transurethral resection at the Clinic for Urology of the University Hospital Schleswig-Holstein (UKSH). Informed written consent was obtained from all volunteers and patients before including them in the retrospective study. The consent from the patients was given preintervention. This study was conducted in accordance with the Declaration of Helsinki and approved by the local ethics committee of the Medical Faculty, Kiel University (#A110/99).

### 2.2. Study Design

The urine from healthy volunteers was studied for DNA concentration under different periods of storage time, gender differences, and time-of-day sampling differences. The urine from bladder cancer patients was screened for cancer mutations.

#### 2.2.1. Healthy Volunteers’ Sample Collection and Storage

Pooling of urine DNA from healthy volunteers was performed based on gender and time-of-day of collection. The collection was conducted for three consecutive days where morning (10 a.m.) and afternoon (3 p.m.) urine was collected separately in sterile, sealed 100 mL Sarstedt urine collection cups. Pooled samples were mixed homogeneously before isolation. Pooled samples from day one were aliquoted into four 50 mL tubes. One aliquot was used for immediate DNA isolation after collection. The remaining aliquots were stored at −20 °C for 1 week, 5 weeks, and 9 weeks before DNA isolation. Isolation for each aliquot was done in triplicates.

#### 2.2.2. Patients’ Sample Collection and Storage

Preoperative and postoperative urine samples were collected from the cancer patients during the visit in sterile, sealed 100 mL Sarstedt urine collection cups. They were filled with 60 mL at most and immediately stored at −20 °C in the Department of Urology and transferred within one week to the Institute of Clinical Molecular Biology, Kiel.

Formalin-fixed, paraffin-embedded (FFPE) tumor samples were prepared by the Department of Pathology of UKSH. FFPE tumor samples were staged and graded by board-certified surgical pathologists, and clinical patient data were obtained by the urologists at the Department of Urology of UKSH.

### 2.3. DNA Isolation

#### 2.3.1. DNA Isolation from Urine Samples

Prior to urine DNA isolation, samples were first equilibrated to room temperature. Once thawed, each sample was mixed homogeneously, 10 mL of urine was aliquoted, and cells were subsequently pelleted to separate cells from other debris and components in urine (Figure S1). The supernatant was then discarded, and the remaining cell pellet was used for DNA isolation using the QIAamp^®^ DNA Micro Kit (Qiagen, Hilden, Germany) according to the manufacturer’s protocol for purification of genomic DNA from urine. The DNA was eluted in 30 µL of AE buffer from the kit. DNA concentrations were measured using the Qubit 2.0 Fluorometer with the Qubit Broad Range dsDNA assay (ThermoFisher Scientific, Waltham, MA, USA). DNA integrity and fragment sizes were analyzed using the 4150 TapeStation System (Agilent, Santa Clara, CA, USA).

#### 2.3.2. DNA Isolation from FFPE Samples

FFPE tumor tissue samples from the surgeries were available for 10 of the 12 patients. Dissected tumor areas were identified and marked on hematoxylin–eosin (H&E)-stained sections prepared from each FFPE sample. Genomic DNA was isolated from 10 μm sections of tumor FFPE samples using the GeneRead DNA FFPE Kit (Qiagen #180134) and Deparaffinization Solution (Qiagen) according to the manufacturer’s protocol. The isolated DNA was kept at 4 °C prior to sequencing. DNA concentrations were measured at room temperature using the Qubit 2.0 Fluorometer with the Qubit Broad Range dsDNA assay (ThermoFisher Scientific). DNA integrity and fragment sizes were analyzed using the 4150 TapeStation System (Agilent).

### 2.4. Sequencing

#### 2.4.1. Next-Generation Sequencing of Urine DNA

Sequencing libraries were constructed from 100 ng of urine DNA using the Nextera^®^ Flex for Enrichment Library Preparation Kit (Illumina, San Diego, CA, USA) according to the manufacturer’s protocol with the following specifications. Amplification of tagmented DNA was performed with 9 PCR cycles. The pooling of preenriched libraries and subsequent steps were performed following the protocol for 250 ng per preenriched library. Amplification of the enriched library was performed with 12 PCR cycles.

Hybridization capture of the libraries was performed in one pool of 22 libraries, using the xGen^®^ Pan-Cancer Panel v1.5 (Integrated DNA Technologies, Coralville, IA, USA) consisting of 7816 probes with 127 gene targets, which are significantly mutated genes identified by TCGA across 12 tissue types [21]. NGS was performed on NovaSeq 6000 (Illumina) with 2 × 150 bp paired-end reads to a median coverage of 200×. The fastq sequencing files were archived at the European Genome-Phenome Archive (EGA) under study accession ID EGAS00001005758. The EGA is subject to the EU’s General Data Protection Regulation, and access to the data may be applied for, which is subject to a data-access agreement with project description and ethics board approval.

#### 2.4.2. Sanger Sequencing for Validation

Sanger sequencing was performed for validation of mutations obtained from NGS and sample swap quality control. Sample-specific variants with high tumor allele frequency were selected for Sanger sequencing. Primer pairs were designed using Primer3Plus [22]. The specificity of the primers was validated using the Basic Local Alignment Search Tool (BLAST) [23]. The primer sequences can be found in Table S1.

PCR amplification was conducted using AmpliTaq^®^ Gold (ThermoFisher Scientific) according to the manufacturer’s protocol. PCR products were analyzed by gel electrophoresis using QIAxcel^®^ ScreenGel software (Qiagen). PCR product clean-up was conducted prior to sequencing using FastAP™ (ThermoFisher Scientific) according to the manufacturer’s protocol. Sequencing was conducted using the BigDye™ Terminator v1.1 Cycle Sequencing Kit (ThermoFisher Scientific) on the Applied Biosystems^®^ 3730 DNA Analyzer (Applied Biosystems, Waltham, MA, USA) according to the manufacturer’s protocol.

#### 2.4.3. 16S Sequencing

DNA was extracted from urine samples using the QIAamp DNA mini kit automated on the QIAcube (Qiagen) according to the instructions of the manufacturer’s protocol. Extracted DNA was stored at −20 °C prior to PCR amplification. Blank extraction controls were included during the extraction of samples. For sequencing, variable regions V1 and V2 of the 16S rRNA gene within the DNA samples were amplified using the primer pair 27F-338R in a dual-barcoding approach according to Caporaso et al. [24]. Three µL of urine DNA was used for amplification. PCR products were verified using the electrophoresis in agarose gel on QIAxcel Advanced System (Qiagen) and normalized using the SequalPrep Normalization Plate Kit (ThermoFischer Scientific). Subsequently, PCR products were pooled in equimolar amounts and sequenced on the Illumina MiSeq with v3 2 × 300 bp paired-end reads. Demultiplexing after sequencing was based on zero mismatches in the barcode sequences. Obtained raw sequencing data were processed using the DADA2 workflow for big datasets, resulting in abundance tables of amplicon sequence variants (ASVs) [25] with customized settings (https://github.com/mruehlemann/ikmb_amplicon_processing/, accessed on: 28 January 2022). The web-based software MicrobiomeAnalyst was used for downstream analyses of microbiota data [26].

### 2.5. Bioinformatic Analysis

Raw sequencing data were demultiplexed and aligned to the Genome Reference Consortium Human Build 37 (GRCh37/hg19) using BWA and GATK v4.1.0.0 within the Exome-seq Pipeline from the IKMB Bioinformatics Platform (https://github.com/ikmb/exome-seq, accessed on: 15 August 2020) [27].

The variant calling and analysis was conducted with GensearchNGS v4.4 (Phenosystems) [28]. Filtering was applied to the mutation calls: variant balance bigger than 0, position balance bigger than 0, minor allele frequency smaller than 0.01, type worse than synonymous, and not equal to locally known variants. The locally known variants are recurring artifacts obtained from the analysis of 53 fresh frozen normal tissue samples from colon cancer patients available under the study accession ID EGAS00001004108 [29].

To obtain tumor-related variants, the variants were filtered for those present in preoperative samples and absent in postoperative samples. Technical validation was performed on the filtered variants by visualizing the variants individually in the GensearchNGS viewer to remove artifacts based on specific discrimination criteria which include (1) excluding variants with alignment or sequencing errors, (2) excluding variants with less than 5% allele frequency with either an in-frame insertion or deletion (indels) or single-nucleotide indels in repeat regions, (3) excluding germline variants with very high allele frequency, and (4) excluding variants whose sequences result in multiple similarities using The Basic Local Alignment Search Tool (BLAST) [23].

Validated variants were then checked across databases for clinical annotations to filter out benign mutations. Considerations for inclusion included (1) clinical significance in databases including NCBI ClinVar [30], Catalogue of Somatic Mutations in Cancer (COSMIC) v91 [31], International Cancer Genome Consortium (ICGC) PanCancer dataset release 28 [32], TCGA release 24, and Integrative Onco Genomics (intOGen) [33], (2) population frequency in GnomAD v2.1.1, and (3) computational verdict based on multiple computational prediction tools available on Varsome, which include DANN, DEOGEN2, EIGEN, FATHMM-MKL, MVP, MutationAssessor, MutationTaster, PrimateAI, REVEL, and SIFT [34].

Sample-pairing quality control was conducted by validating preoperative and postoperative sample pairing based on common single-nucleotide polymorphisms (SNPs) among the variants found within the patient cohort as covered by the IDT Pan-Cancer Panel v1.5 (Table S2).

### 2.6. Prostate Cancer Tissue Analysis

The prostate cancer tissues from the two patients with incidental prostate cancer (iPCa) were examined for *BRCA1* mutations in the routine pathological procedures using tumor-bearing FFPE tissue sections and the AmpliSeq for Illumina BRCA Panel (Illumina) according to the manufacturer’s protocol.

### 2.7. Statistical Analysis

For 3 or more group comparisons (Figure S2A,B), a one-way analysis of variance model (ANOVA) was performed, and a Dunnett adjustment for multiple comparisons was used. Data shown as bar graphs represent the mean and standard error of the mean (s.e.m.) of a minimum of 3 biological replicates. All *p*-values reported are two-sided and considered as significant if < 0.05. ns > 0.05, * < 0.05, ** < 0.01, *** < 0.001, **** < 0.0001. For two-group comparisons (Figure S2C–F), a *t*-test with Welch’s adjustment was performed. Error bars represent s.e.m.

## 3. Results

**Figure 1:**
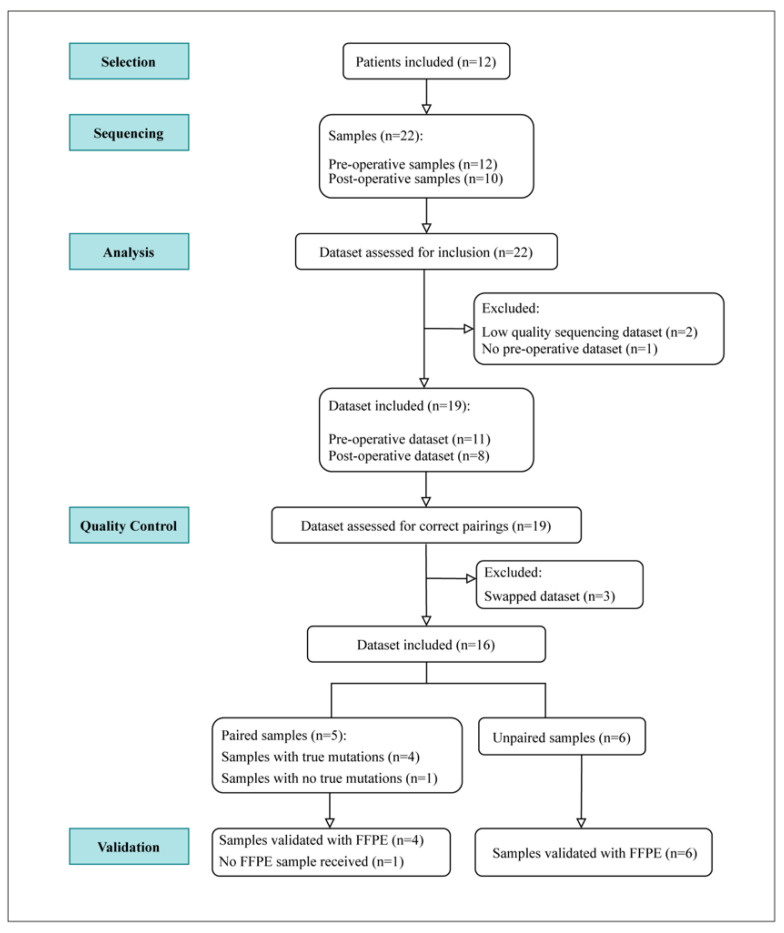
Experimental setup. Twelve patients were included, resulting in 22 urine samples—12 of which were preoperative and 10 postoperative urine samples. Following DNA isolation and subsequent sequencing, the resulting DNA dataset was assessed for inclusion. Datasets from 19 samples were included and later assessed for correct pairings. Three postoperative datasets were excluded due to potential swap or incorrect pairings. This resulted in five validated paired samples and six unpaired samples. Following quality control, 10 samples were validated with FFPE tumor samples.

The evaluation approach used in this study is represented as a flow diagram in Figure 1. In total, 22 urine samples from the 12 patients were isolated for DNA and then sequenced. Twelve were preoperative samples, and ten were postoperative samples. The sequencing datasets were then assessed for inclusion resulting in the exclusion of 3 datasets and the inclusion of 19 datasets. For quality control, each sequencing dataset was tested for the correct pairing of preoperative and postoperative data. As a result, three datasets were excluded due to a potential research laboratory sample swap of the doubly pseudonymized urine specimens. From the remaining datasets, five samples had correct pairs, and six samples had either missing or unavailable pairs. Among the paired samples, one sample showed no true mutations. Validation with FFPE tumor tissues was then conducted for 10 samples.

### 3.1. Urine DNA Yield in Healthy Pools after Different Storage Periods

The DNA yield from healthy volunteers’ urine showed high variability in concentration across different collection days and times (Table S3). The DNA yields for the female pool across the three daily collection days ranged from 30.6 ng/µL to 107 ng/µL for the daytime collection and from 38.6 ng/µL to 116 ng/µL for the evening collection. For the male pool, the DNA yields across the three daily collection days ranged from 1.7 ng/µL to 2.9 ng/µL for the daytime collection, and from 1.6 ng/µL to 3.8 ng/µL for the evening collection. Comparing both pools on average, the female pool at 68 ng/µL showed a 27-times-higher DNA concentration compared to the male pool with an average of 2.5 ng/µL.

Pooled urine samples from the collection on day 1, which were stored for different periods of time, showed a pattern of decreasing DNA concentration as the freezing period increased. After one week of storage, the urine DNA concentration was reduced by approximately 20% on average across all pools and declined to 8% after 5 weeks and 6% after 9 weeks, as shown in Figure S2A,B. Overall, urine collected from females had significantly higher values compared to that of males (Figure S2C–F).

### 3.2. Patient Cohort

The clinical characteristics of the patient cohort are summarized in Table 1. A summary of the samples collected from each patient is shown in Tables S4 and S5.

### 3.3. Variant Analysis

In total, 114 mutations were found within the cohort with 90 variants predicted as either pathogenic, likely pathogenic, or having uncertain significance according to the ACMG classification rules (Table S6). For each sample except U6, at least one variant was present in a gene related to bladder cancer. In 75% of the samples analyzed, the highest tumor allele fraction was in bladder-cancer-associated genes (Figure S3). These genes include *FGFR3*, *RB1*, *APC*, *ARID1A*, *NCOR1*, and *KDM6A*.

Within the patient cohort, 47 genes were found to be mutated. Twenty-nine of the genes were found to be within the top 200 mutated genes based on The Cancer Genome Atlas—Bladder Cancer (TCGA-BLCA) projects [35], and 17 of the genes were marked as driver genes in bladder cancer based on intOGen [33]. Fifteen out of the eighteen genes with more than 1 mutation were bladder-cancer-associated genes (Figure S4). *TP53* was the most frequently mutated gene with a total of 10 variants found in the cohort. This was followed by *BRCA1* with six variants and *ATM* and *FGFR3* with five variants each.

**Figure 2:**
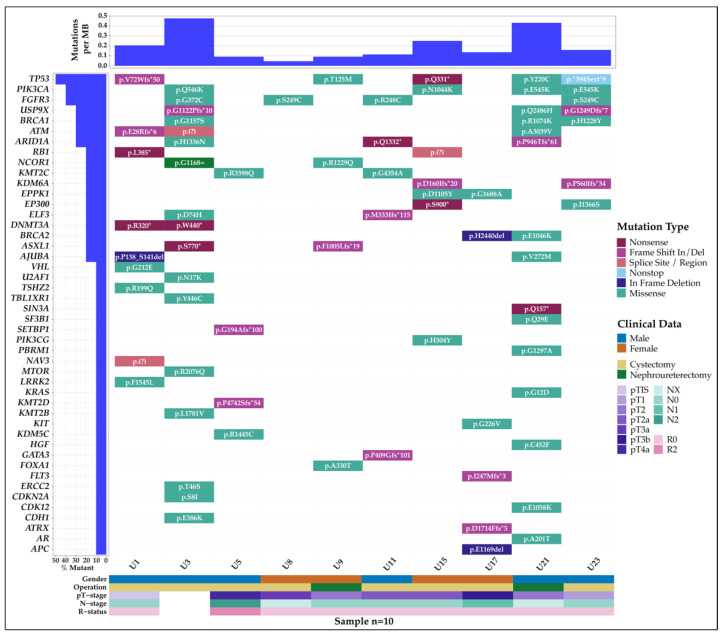
Mutational landscape of the patient cohort. Each column represents a patient and their mutated genes, gender, operation, and pTNR staging. Samples U1, U3, U5, U11, and U17 are samples without postoperative urine pairs. No tumor was found in U3 after neoadjuvant chemotherapy. Patients U3 and U23 were found to have incidental prostate cancer.

The mutational landscape of this cohort is summarized in Figure 2, which includes the pathologic staging of the tumor (T), regional lymph nodes (N), and the presence of residual tumor (R). The majority of the mutations were typed as missense mutations. Among the eight cystectomy patients, the most frequently mutated gene was *FGFR3*, which was found four patients—all of which were missense mutations. Patient U3 was found to have no tumor in the bladder after neoadjuvant chemotherapy but was found to have incidental prostate cancer (iPCa). Patient U3 also showed the highest mutational burden with multiple mutations among frequently mutated genes. Patient U23 was also found to have iPCa.

For the iPCa patients, the prostate cancer tissue was examined for the BRCA mutations detected in their preoperative urine samples; however, these mutations were not detected in the prostate cancer tissue.

### 3.4. Concordance with Tumor Tissues

To validate the results obtained from NGS, sequencing of the patients’ tumor FFPE samples was performed, targeting 2 quality control variants and 19 sample-specific variants. All samples were concordant for the two quality control variants. Out of the 19 sample-specific variants, 14 were concordant with the urine NGS results. Exceptions to this include samples U15 in the *TP53* variant and U17 in the *ATRX* and *KIT* variants—all of which were low-frequency variants from female patients’ urines. There were also two instances where the primer did not capture the intended target region. Results of the Sanger sequencing are summarized in Table S7.

## 4. Discussion

### 4.1. Preoperative Urine Samples Harbor Tumor-Specific Mutations from Bladder Cancer

Analysis of the preoperative samples’ sequencing datasets shows that in most patients, the number of variants was higher compared to their corresponding postoperative samples. We observed that among the variants, tumor-specific mutations from bladder cancer could be found in almost all patients. Although the number of patients evaluated in this study was small, our observation is in line with Springer et al., who detected genetic abnormalities in over 70% of their bladder cancer patient cohort [19]. As a proof of principle, our findings underscore the utility of urine liquid biopsy for detecting tumor mutations. Only one patient, U6, did not have any relevant mutations. This can be explained by the very low sequencing coverage (Figure S3) for U6′s preoperative sample. Of note, we also observed the same variants from our NGS datasets in the majority of the patients’ FFPE tumor tissue samples. However, there are exceptions in some samples, notably U15 and U17, which displayed only low-frequency variants in the urine DNA that were not detected in the tissue. This discrepancy may be due to tumor heterogeneity as the region obtained from the FFPE sample may not be representative of the whole tumor landscape of the patients.

### 4.2. Matching Pre- and Postoperative Urine Eliminates Germline Mutations and Provides a Strategy to Assess Remaining Tumor Burden in a Patient

While pathology testing is often carried out using tumor samples only, we observed that utilizing nontumor samples helps to pinpoint tumor-specific variants in cases of doubt. As the majority of the patients in our cohort underwent surgery such as cystectomy and nephroureterectomy, both leading to the complete removal of the tumor, the postoperative urine sample would supposedly only contain normal, nontumor cells. Sequencing the DNA from the postoperative sample allowed for the identification of patient-specific germline variants specific for each patient. Filtering these variants out from the preoperative datasets narrowed down the number of variants to tumor-specific ones only. Additional filtering for potential sequencing or alignment artifacts was performed to eliminate variants that were recurrent with a high frequency in a panel of 54 healthy DNA samples sequenced with the same method but not listed in public databases. These resulting tumor-specific mutations can be used as biomarkers at an early stage to identify factors that may contribute to disease progression, recurrence, or resistance. Our results demonstrate the potential of our method as a surveillance tool in a disease with a high rate of recurrence [36,37]. Looking beyond surveillance, in the context of muscle-invasive bladder cancer, preoperative urine liquid biopsy has the potential to aid the selection of chemotherapy and immunotherapy appropriate for each patient. Further studies are warranted to explore this potential to provide optimal management to patients. In terms of practicality, blood taken at the time of surgery would be an alternative to postoperative urine as a substance that can be used to identify and eliminate germline variants in the preoperative urine.

### 4.3. Technical Consideration in Urine Liquid Biopsy

We also found some important technical aspects, which merit consideration in the handling of urine samples. In healthy volunteers, the DNA concentration decreased with increasing storage time of the urine samples at −20 °C (Figure S2A,B). This phenomenon was also observed in our patient cohort, without generally compromising the detection of tumor mutations, which is in line with a study reported by Bali et al. [38].

However, we did observe a reduction in the total detectable number of variants after a prolonged storage time, although the prominent tumor-related variants were still detectable (data not shown). Thus, storage primarily affects the detection rate of low-frequency mutations. Therefore, we recommend that the period between urine collection and DNA isolation should be as short as possible. Ideally, the DNA isolation should take place immediately after the urine collection in order to ensure the optimal DNA concentration for downstream analyses.

Interestingly, we also noticed in our healthy volunteers a high variability of DNA amounts in relation to the day and time of collection (Table S3). Across each sampling time point, we found that the second-morning urine contained the largest amount of DNA, which provides sufficient material for downstream analysis. This finding could serve as a guide for the optimal time point to collect urine samples, which is important for longitudinal monitoring purposes. This is particularly relevant in patients who underwent nephroureterectomy and thus have a high relapse rate [39,40].

Similar to previous studies, we observed that samples taken from women yielded significantly higher DNA concentrations when compared with those from men (Table S3, Figure S2) [20,41,42,43,44]. To exclude potential bacterial contamination or overgrowth in females, we performed 16S rDNA amplification and sequencing (Figure S5), which revealed rather lowered overall bacterial richness and abundance in urine samples from females compared to those from males (Figure S5A–C). In addition, we performed compositional analyses of all samples and found no significant differences in bacterial composition between females and males, besides some gender-specific alterations that have been described before (Figure S5D,E) [45]. The higher amount of human DNA in female urine than in male urine has been suggested to be due to physiological and anatomical differences, for example, females have a higher number of epithelial cells in addition to genital secretions [42,46]. This characteristic is important to take into consideration, especially in clinical settings where a sufficient amount of DNA is required for downstream analysis. Therefore, it is important to collect a sufficient volume of urine to compensate for samples with extremely low concentrations such as those from men. Although it was not a point of investigation in our paper, bladder cancer patients after cystectomy allow for the exclusion of anatomical differences and possible hormonal differences as most female patients are postmenopausal. Another study in those patients and healthy individuals would help define the factors affecting the DNA yield in urine.

In the context of the SARS-CoV-2 pandemic, the medical system is challenged by supply problems of essential products, frequent changes to regulations, overloaded intensive care units, and limited clinical staff due to infections and quarantine. On the other hand, patients are faced with significantly delayed procedures for diagnosis, treatment, and surveillance. This leads to delays in intervention, a higher frequency of cystoscopy usage, increased time to treatment, and reduced compliance to maintenance strategy as reported by Ferro et al. [47]. These observations are consistent with those of other medical institutions [48,49] and are also seen in the care of other cancers [50,51,52]. Although long-term overall implications are yet to be assessed, further delays and impediments in the management procedures would only increase the burden to patients and hospitals. To mitigate these issues, urine liquid biopsy may in the future serve as a strategy, considering the ease of obtaining, submitting, and processing the samples in clinics by reducing the time, number of staff, and cost needed while minimizing their potential exposure to SARS-CoV-2.

### 4.4. Study Limitations and Future Outlook

In addition to our findings, there are limitations to our study which can be taken into consideration for subsequent future research. Due to the ongoing pandemic, challenges include the recruitment of patients—which limited some depth in our analysis. As a future outlook, our results justify follow-up studies with larger cohorts of patients representing the different subtypes of bladder cancer.

## 5. Conclusions

We have investigated and concluded the benefits of utilizing urine in the detection and management of bladder cancer and highlighted the sectors that could benefit the most from its routine use. Collectively, the analysis of urine DNA sequencing datasets with a large 127-gene PanCancer panel is able to provide a molecular characterization of a patient’s urothelial cancer condition. In addition, we observed that urine is a robust sample with advantageous characteristics and is suitable for use in clinics. Thus, our study is in line with others that suggest that urine liquid biopsies should be regularly incorporated into bladder cancer management. However, whether urine DNA sequencing with a large gene panel should be used as an alternative to the current standard of care or as a complementary method remains to be elucidated. Although the cohort size was limited in this study, the results favorably support the utility of urine liquid biopsy. The ability to monitor specific genetic mutations would help complement or decrease the need for invasive diagnostic tools that are currently being used in bladder cancer management. This will provide the basis to further investigate the role of specific genetic mutations in both neoadjuvant and adjuvant chemotherapy responses, which will open the door to individualized chemotherapy treatment for urothelial cancer.

To conclude, we believe this study serves as preliminary evidence supporting the utility of our urine sequencing approach in the management of bladder cancer patients, and therefore this method deserves further investigation.

## Figures and Tables

**Table 1:** Clinical data of cancer patients in the cohort.

Characteristics	n
Total patients	12
Male	7
Female	5
Age (in years)	
Mean	70.7
Median	72
Range	52–81
Type of operation for tumor resection	
Cystectomy	9
Nephroureterectomy	2
Transurethral resection of bladder tumor (TURBT)	1
Immunohistochemistry markers	(positive/negative)
AMACR	1/5
CK7	1/5
CK8	1/5
CK20	5/1
GATA3	2/4
p63	1/5
No available data	6
Pathologic staging of the tumor (pT-stage)	
Tis	3
T1	1
T2	2
T2a	2
T3a	1
T3b	1
T4a	1
No tumor found ^1^	1
Regional lymph nodes (N-stage)	
NX	4
N0	6
N1	1
N2	1
Presence of residual tumor (R-status)	
R0	11
R2	1
Lymphovascular invasion (LVI)	
Positive	3
Negative	9
Vascular invasion (VI)	
Positive	3
Negative	9
Perineural invasion (PNI)	
Positive	1
Negative	11

Notes: Tis: carcinoma in situ. T1–T4: size or extent of the primary tumor. NX: lymph nodes cannot be assessed. N0: no regional nodal metastasis. N1: involvement of 1–3 regional nodes. N2: involvement of 4–6 regional nodes. R0: no residual tumor. R2: macroscopic residual tumor. ^1^ No tumor found after neoadjuvant chemotherapy.

## Data Availability

The fastq sequencing files were securely archived at the European Genome-Phenome Archive (EGA) under study accession ID EGAS00001005758.

## Supplementary Materials

The following are available online at https://www.mdpi.com/article/10.3390/cancers14040969/s1. Figure S1: representative images of the typical pelleted sediments and DNA electropherogram of urine samples, Figure S2: DNA yield in representative healthy pools at different storage periods, Figure S3: sequencing depth and highest tumor allele fraction, Figure S4: most frequently mutated genes found in the patient cohort, Figure S5: 16S rDNA amplification and sequencing results of urine samples. Table S1: list of the 22 primer pairs used for Sanger sequencing and their forward and reverse sequences, Table S2: list of common SNPs obtained from the patient cohort. Table S3: the average DNA concentration in different pools of healthy volunteers, Table S4: summary of the results from TapeStation and Qubit quantification of isolated DNA from each sample, Table S5: immunohistochemistry markers for each patient, Table S6: list of nonbenign true mutations found in the cohort, and Table S7: summary results of Sanger validation with FFPE samples.
