# Supplementary material for: Detection of Cancer Mutations by Urine Liquid Biopsy as a Potential Tool in the Clinical Management of Bladder Cancer Patients
Source: Cancers (Basel). 2022 Feb 15;14(4):969. doi: 10.3390/cancers14040969 (PMC8870091; doi:10.3390/cancers14040969)
Supplement: Supplementary file 1 [file cancers-14-00969-s001.zip › cancers-1533743-Supplementary Materials.pdf]

Supplementary materials

Detection of Cancer Mutations by Urine Liquid Biopsy as a Potential Tool in the Clinical Management of Bladder Cancer Patients

Nurul Khalida Ibrahim[1,2], Ahmed Eraky[3], Jan Eggers[3], Tim Alexander Steiert[2], Susanne Sebens[4], Klaus-Peter Jünemann[3], Alexander Hendricks[5], Corinna Bang[2], Martin Stanulla[1], Andre Franke[2], Claudius Hamann[3], Christoph Röcken[6], Norbert Arnold[2†], Laura Hinze[1†], Michael Forster[2†*]

1 Department of Pediatric Hematology and Oncology, Hannover Medical School, Hannover 30625, Germany; ibrahim.nurul@mh-hannover.de (N.K.I.); hinze.laura@mh-hannover.de (L.H.); stanulla.martin@mh-hannover.de (M.S.)

2 Institute of Clinical Molecular Biology, Kiel University, Kiel 24105, Germany; t.steiert@ikmb.uni-kiel.de (T.A.S.); a.franke@mucosa.de (A.F.); c.bang@ikmb.uni-kiel.de (C.B.), norbert.arnold@uksh.de (N.A.); m.forster@ikmb.uni-kiel.de (M.F.)

3 Department of Urology, University Hospital Schleswig-Holstein Campus Kiel, Kiel 24105, Germany; Ahmed.Eraky@uksh.de (A.E.); Jan.Eggers@uksh.de (J.E.); Klaus-Peter.Juenemann@uksh.de (K-P.J.); Claudius.Hamann@uksh.de (C.H.)

4 Institute for Experimental Cancer Research, Kiel University and University Hospital Schleswig-Holstein Campus Kiel, Kiel, Germany; susanne.sebens@email.uni-kiel.de (S.Se.)

5 Department of General Surgery, University Medicine Rostock, Rostock 18057, Germany; alexander.hendricks@med.uni-rostock.de (A.H.)

6 Department of Pathology, University Hospital Schleswig-Holstein Campus Kiel, Kiel 24105, Germany; Christoph.Roecken@uksh.de (C.R.)

* Correspondence: m.forster@ikmb.uni-kiel.de; Tel.: +49 431 500 15136

† These authors are shared last authors.

Supplementary Materials

Supplementary Figures

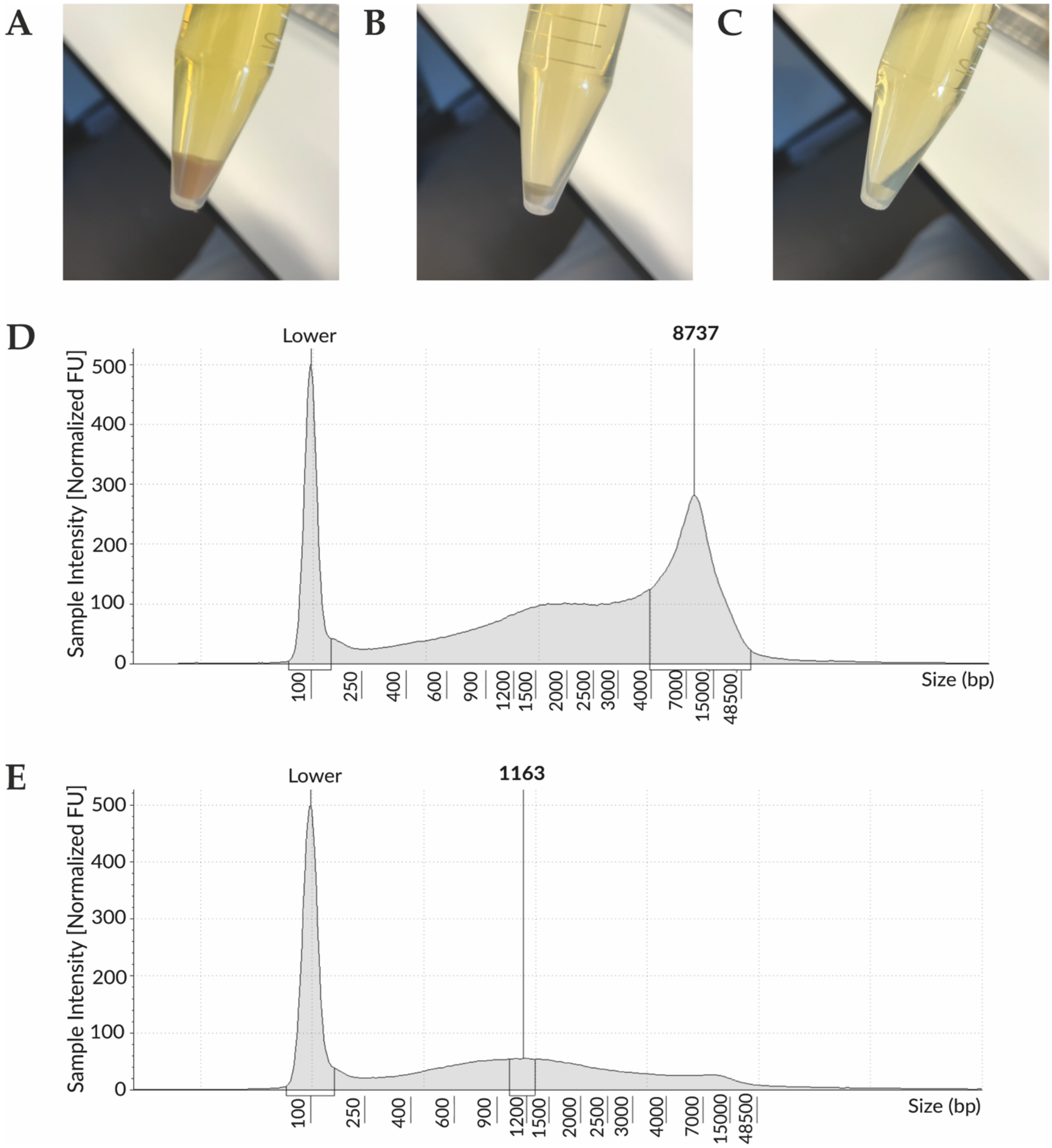

Figure S1. Representative images of the typical pelleted sediments and DNA electropherogram of urine samples**.** Pelleted sediments either appeared **a)** brown or dark red in color typically seen in pre-operative samples, **b)** two-layered with a brown or dark red layer above a clear or white layer or **c)** clear or cloudy layer typically seen in post-operative and healthy volunteer samples. Pellet A can be described as very dense and packed whilst pellet C can be described as light and delicate with a feathery appearance. Example of average electropherograms obtained from different samples: **d)** healthy urine sample, **e)** patient urine sample. Peak intensity signifies the majority DNA fragment size found within each sample.

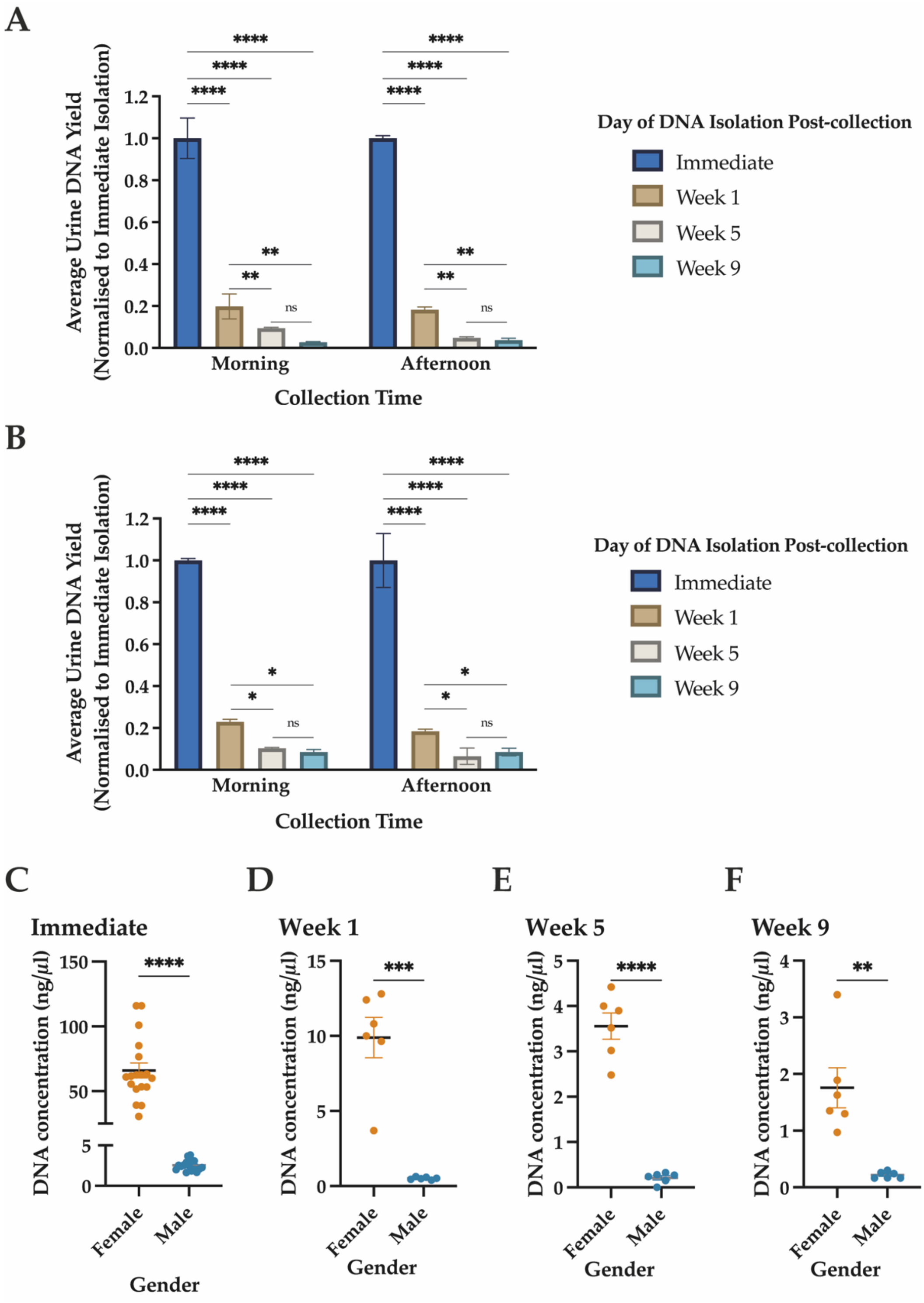

Figure S2. DNA yield in representative healthy pools at different storage periods. **a)** Female sample pool (n= 5); **b)** Male sample pool (n= 5). Both graphs show decreasing concentrations as the frozen storage period increases. One-way ANOVA was performed and a Dunnett adjustment for multiple comparisons were used to compare the significance of differences in DNA yield across samples after different storage period post collection. Data shown as bar graphs represent the mean and standard error of the mean (s.e.m) of a minimum of 3 biologic replicates. **c-e)** Comparison between genders from all pooled samples collected. DNA isolated **c)** immediately after collection for all 3 days of collection; **d)** One-week-post collection; **e)** Five-week-post collection; **f)** Nine-week-post collection. A t-test with Welch's adjustment was performed to compare the significance between female and male DNA yield. Error bars represent s.e.m. All p-values reported are two-sided and considered as significant if <0.05. ns > 0.05, * <0.05, ** < 0.01, ***<0.001, ****<0.0001.

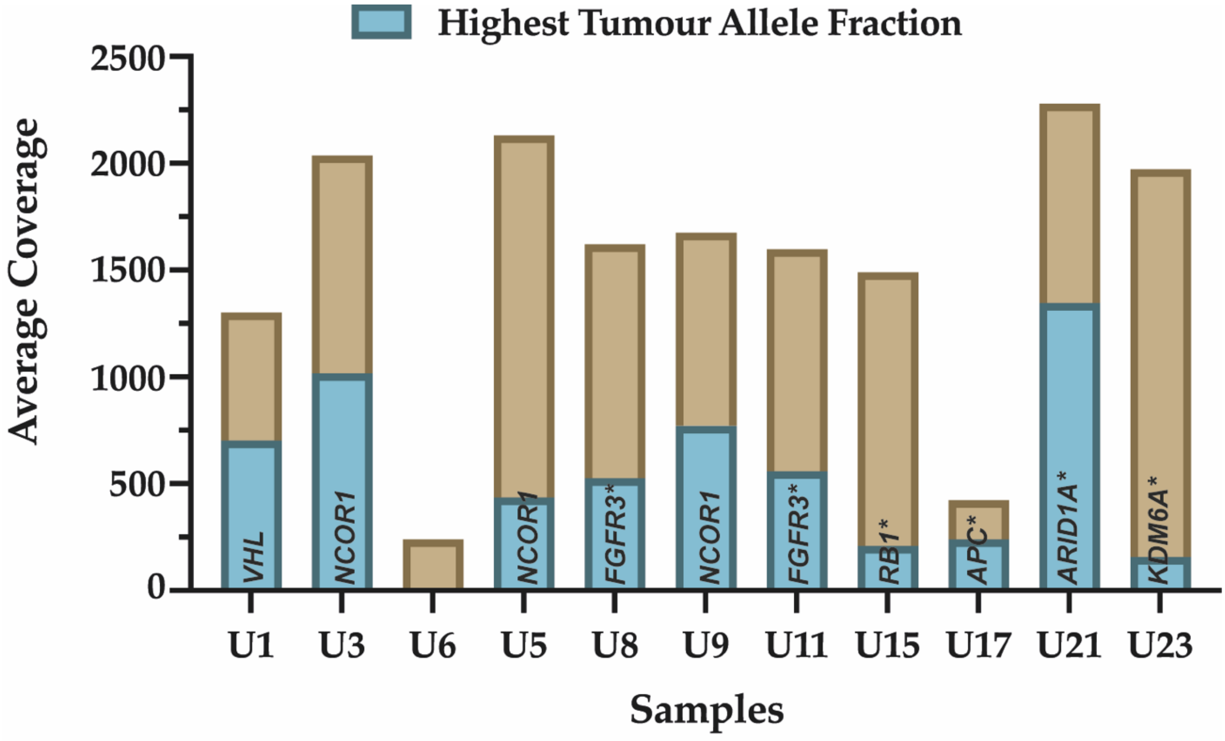

Figure S3. Sequencing depth and highest tumor allele fraction. The coverage shown is the average coverage of the pre-operative sample for each patient. The highest tumor allele fraction is calculated as the average coverage multiplied by the highest tumor allele frequency. Genes with the highest tumor allele frequency in each sample are labeled on each bar. Genes marked with an asterisk (*) have variants classified as pathogenic or likely pathogenic according to the ACMG variant classification. Samples U1, U3, U5, U9, U11, and U17 only have pre-operative sequences and therefore may contain germline variants within their total.

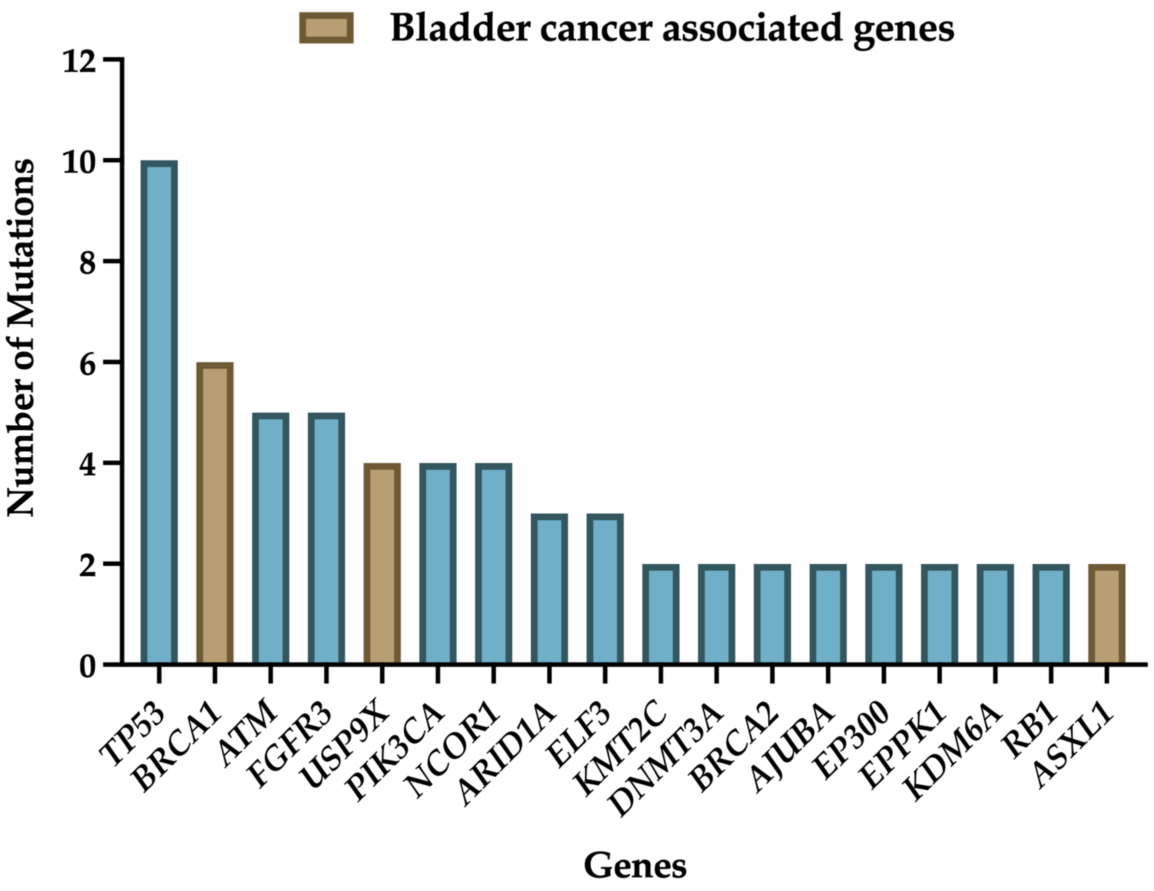

Figure S4. Most frequently mutated genes found in the patient cohort. Gene names and the number of mutations are shown. Other mutated genes with only one occurrence are *APC, AR, ATRX, CDH1, CDK12, CDKN2A, CHEK2, ERCC2, FLT3, FOXA1, HGF, KIT, KMT2B, KMT2D, KRAS, LRRK2, MTOR, NAV3, PBRM1, PIK3CG, SETBP1, SIN3A, TBLX1R1, TSHZ2, U2AF1,* and *VHL.*

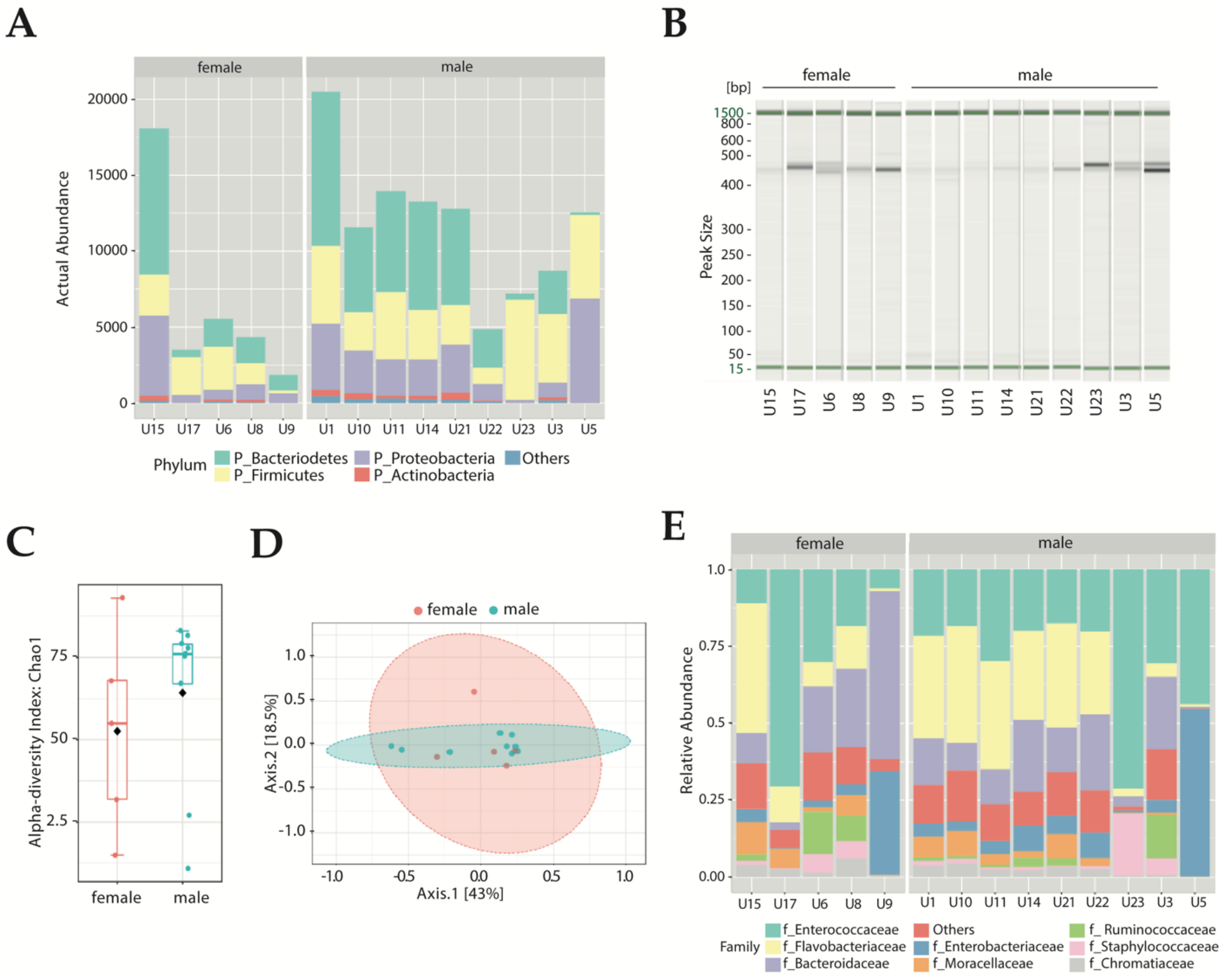

Figure S5: 16S rDNA amplification and sequencing results of urine samples. **a)** Absolute abundances of sequencing reads after 16S rDNA sequencing and processing of raw data using DADA2 workflow; **b)** 16S-PCR products of urine DNA samples; **c)** Microbiota alpha diversity was estimated using Chao1 index. These measures were calculated on rarefied ASV table sequences per sample; **d)** Beta diversity was calculated with Bray-Curtis dissimilarity and Principle Coordinate Analysis was applied to visualize the level of dissimilarity of samples; **e)** Overview of the microbial community composition of the most abundant bacterial families for all samples analyzed.

Supplementary Tables (Tables in excel file)

Table S1. List of the 22 primer pairs used for Sanger sequencing and their forward and reverse sequences.

Table S2. List of common SNPs obtained from the patient cohort.

Table S3. The average DNA concentration in different pools of healthy volunteers.

Table S4. Summary of the results from TapeStation and Qubit quantification of isolated DNA from each sample.

Table S5. Immunohistochemistry markers for each patient.

Table S6. List of non-benign true mutations found in the cohort

Table S7. Summary results of Sanger validation with FFPE samples.
